# Characterization and estimation of heterogeneous spatial autocorrelation in spatial autoregressive models

**DOI:** 10.1371/journal.pone.0327316

**Published:** 2025-07-01

**Authors:** Jing Zhao, Yue Pu

**Affiliations:** 1 Army Logistics Academy, Chong Qing, China; 2 Beijing Police College, Bei Jing, China; Cairo University, EGYPT

## Abstract

Spatial Autoregressive (SAR) models are widely used to analyze interactions among regions. However, the traditional model assumes a constant spatial autocorrelation coefficient, which fails to effectively capture spatial heterogeneity. To address this issue, we propose proposes a novel Spatial Single-Index Varying Coefficient Autoregressive (SSIVCAR) model. By introducing a single-index varying coefficient function, this model allows the spatial correlation strength to dynamically change with the characteristics of spatial units, thereby more accurately capturing spatial dependence relationships. To estimate the model parameters, we combine spline methods with two-stage least squares, and we assess the model’s performance under finite sample conditions under Monte Carlo simulations. The simulation results show that the proposed model performs significantly better in capturing spatial heterogeneity and improving estimation accuracy. Finally, the model is applied to analyze the impact of digital economy development on environmental quality, and find that it has significant heterogeneous effects across different regions. This study provides a new framework for analyzing complex spatial dependence structures and offers valuable insights for regional governance policies.

## Introduction

Spatial statistics is a distinct and well-established discipline focused on analyzing spatially referenced data. In recent years, it has played an increasingly important role in the study of environmental economics, regional development, and related fields. A central feature of spatial data is spatial dependence, which captures the interrelatedness of observations across geographic space. For example, Anselin [1] pointed out that the diffusion of air pollutants often crosses administrative boundaries, leading to a high correlation in environmental quality between regions; LeSage [2] further emphasized that ignoring spatial dependence may lead to significant bias in estimating policy effects. However, in addition to spatial dependence, spatial heterogeneity between regions, such as differences in economic structure, industrial layout, and population distribution, also significantly affects model identification and policy formulation.

The traditional Spatial Autoregressive (SAR) model [3], which captures spatial dependence through a fixed parameter ρ, has become the benchmark tool in empirical research due to its simplicity. However, this homogeneity assumption often fails in real-world scenarios. For example, Feng [4] found that in the Yangtze River Delta region, the spatial correlation of industrial pollution emissions is as high as 0.8 due to the close collaboration of industrial chains, whereas in ecologically fragile western regions, the spatial correlation ρ is below 0.3 due to geographic barriers. Ignoring this heterogeneity may lead to an underestimation of the collaborative governance needs in economically developed areas, while overestimating the independent emission reduction potential in less developed areas.

To address this issue, researchers have attempted improvements from multiple perspectives. One approach introduces spatial heterogeneity structures, such as multiscale models [5,6,20] and spatial varying-coefficient models [7–9], by assigning different parameters across regions to capture regional differences [10]. These methods have alleviated the limitations of the homogeneity assumption to some extent, but there are still difficulties in handling high-dimensional feature spaces and capturing dynamic patterns. Another line of research adopted nonparametric or semiparametric methods, attempting to flexibly capture spatial heterogeneity without pre-setting specific functional forms [11]. Although these methods improve model flexibility, they still focus on explaining the relationships and tend to overlook the dynamic variation in ρ. In these methods, spatial effects are often simplified or omitted, making it difficult to fully reveal the differences in spatial dependence between spatial units. Therefore, these methods remain limited in addressing the combined effects of spatial heterogeneity and spatial dependence [12].

To address these limitations, we propose a novel spatial single-index varying coefficient autoregressive (SSIVCAR) model. Unlike the SAR model, SSIVCAR links regional characteristics *u*_*i*_ to the spatial autoregressive coefficient via a nonlinear single-index function $g(u_{i}^{⊤}\alpha )$. This design captures the heterogeneity in spatial correlation and reveals how regional characteristics influence spatial dependence, enabling more accurate modeling of heterogeneous spatial structures. It relaxes the constant correlation assumption in the SAR model, allowing spatial effects to vary across regions. While maintaining the basic structure of the SAR model, the second part still adopts a linear regression model form, preserving the simplicity and interpretability of the model. The model also ensures computational efficiency when dealing with large-scale spatial data by combining spatial correlation with linear covariate relationships. Furthermore, considering the endogeneity issue, we adopt a joint estimation method of spline functions and two-stage least squares (2SLS) to eliminate potential endogeneity bias and improve the accuracy of parameter estimates.

Monte Carlo simulations were conducted to evaluate the performance of the proposed model under finite sample conditions. The results show that the SSIVCAR model outperforms the SAR model in terms of estimation accuracy and robustness. Under conditions of strong spatial heterogeneity, the proposed model significantly improves the reliability of the estimation results. Finally, we apply the SSIVCAR model to examine the impact of digital economy development on environmental quality. The empirical findings indicate that digital economy development has a significant and heterogeneous impact on various types of environmental pollution.

The structure of the following sections is as follows. Sect 2 discusses related works; Sect 3 introduces and derives the SSIVCAR model, including its basic framework and estimation methods; Sect 4 presents Monte Carlo simulations to evaluate the model’s performance under finite samples; Sect 5 provides an empirical analysis, applying the SSIVCAR model to investigate the impact of digital economy development on environmental pollution in China; Sect 6 concludes the paper by summarizing the main findings and suggesting directions for future research.

## Related works

The SAR serves as a foundational framework for analyzing spatial dependence. Its classic form was systematically developed by Anselin [1], characterizing interactions between neighboring units through a spatial lag term. LeSage and Pace [2] further expanded the Bayesian estimation method and developed a spatial econometrics toolbox that is widely used in policy evaluation. To address endogeneity, Kelejian [15] proposed the generalized spatial two-stage least squares (GS2SLS), providing a theoretical basis for instrument variable selection. Elhorst *et al*. [16] developed a dynamic spatial panel model that allows for both temporal and spatial dependence; Lee *et al*. [12] derived the asymptotic properties of the quasi-maximum likelihood estimator (QMLE). In recent years, high-dimensional data analysis has driven SAR models toward sparse modeling. Lam *et al*. [17] proposed a penalized maximum likelihood method suitable for variable selection in high-dimensional settings. Additionally, Ejigu [38] introduced a covariate-dependent weighting matrix that integrates spatial proximity and observed characteristics. This approach challenges the traditional assumption that geographic closeness alone defines neighborhood structure and demonstrates improved model fit in environmental applications. These developments extend the flexibility of SAR-type models by allowing spatial dependence to vary with contextual covariates.

To address the limitations of global spatial models in capturing local heterogeneity, researchers have developed the geographically weighted regression (GWR) framework. GWR performs localized parameter estimation through a spatial kernel function, as systematically outlined by Brunsdon and Fotheringham [18]. Brunsdon *et al*. [19] proposed an adaptive bandwidth selection algorithm that optimizes the kernel range via cross-validation. To enhance model flexibility, Comber *et al*. [20] developed the multi-scale GWR (MGWR), which allows different variables to operate at different spatial scales. With the development of machine learning, Hagenauer [21] integrated GWR with artificial neural networks, while Lu *et al*. [22] proposed a covariate-weighted GWR (CS-GWR) to address dimensionality issues via variable selection. However, GWR methods face two major limitations: First, they rely heavily on geographic distance weights, limiting their ability to model heterogeneity arising from socio-economic factors [14]; Second, their computational complexity grows quadratically with sample size, limiting scalability in big data contexts [23]. To partially address these issues, Ejigu [41] applied Bayesian geostatistical models to analyze modern contraceptive use in Ethiopia. By incorporating individual- and service-level covariates, the study demonstrated how flexible spatial models can better capture social and demographic heterogeneity beyond geographic proximity.

Spatially varying coefficient models (SVCM) have also garnered considerable attention for modeling local structural variation. The original varying coefficient model framework was proposed by Hastie and Tibshirani [24], enhancing model flexibility through localized parameterization. Aquaro *et al*. [10] extended this framework to spatial settings by developing heterogeneous coefficient spatial regression models. Yang [25] proposed instrumental variable quantile estimation with nonparametric spatial weights, and Sun [26] introduced local linear methods to improve estimation efficiency. More recently, Chen *et al*. [27] adopted spline basis functions to approximate varying coefficient surfaces and established corresponding asymptotic theory; Liu [28] developed an adaptive LASSO penalized SVCM for simultaneous variable selection and estimation. However, SVCM methods still face two key challenges: the curse of dimensionality when handling high-dimensional covariates [29], and limited attention to spatial lag endogeneity [30]. Furthermore, most existing approaches assume stationarity in spatial dependence. Addressing this, Ejigu [39] proposed a geostatistical model incorporating covariate-driven nonstationarity in the covariance structure. Simulation results and case studies in disease mapping show that ignoring nonstationarity can lead to biased inference and misestimated uncertainty. Moreover, Ejigu [40] applied spatial models to map malaria risk in Mozambique, identifying social and environmental determinants of childhood infection. These studies underscore the practical relevance of flexible spatial modeling in real-world epidemiological applications.

In the realm of semiparametric modeling, the single-index model (SIM) strikes a balance between flexibility and interpretability by reducing dimensionality. Its theoretical foundation was established by Ichimura [31], who proposed a semi-parametric least squares estimator. Yu and Ruppert [32] introduced penalized spline estimation to control model complexity and mitigate overfitting. In spatial econometrics, Klein *et al*. [33] developed a single-index spatial error model (SI-SEM) to capture nonlinear housing price diffusion mechanisms; Li [34] embedded the single index structure into a spatial lag framework and proposed a semiparametric SAR model; Guan [35] extended this framework to construct a single-index spatially varying coefficient model (SI-SVCM), using splines to approximate locally varying effects. However, existing SIM-based models seldom address heterogeneity in spatial lag parameters or account for endogeneity within a unified framework. These gaps motivate the development of more general spatial semiparametric frameworks capable of capturing complex spatial interaction patterns.

## Model

### Preliminary experiment

The SAR model is widely used in spatial econometrics to capture spatial dependence and autocorrelation. The standard form of the SAR model is given by [1]:

Y=ρWY+Xβ+ε (1)

where *Y* is the dependent variable, *W* is the spatial weight matrix, ρ is the spatial autoregressive coefficient, *X* is the matrix of explanatory variables, β is the regression coefficient, and ε is the error term. The spatial correlation coefficient ρ in the model is assumed to be a constant, representing the overall degree of spatial autocorrelation in the sample.

However, the assumption that ρ is a constant implies that spatial correlation is the same across all samples, which may not hold in practice. In many real-world applications, spatial effects may vary across regions or units. For example, in regional economic studies, the economic behaviors of neighboring regions may be influenced by different spatial effects. Therefore, assuming that all samples share the same spatial correlation coefficient ρ may be overly simplistic and neglect the issue of spatial heterogeneity.

This raises a fundamental question: Should the spatial correlation coefficient be assumed identical across all observations? In other words, can the model be extended to allow observation-specific spatial correlation coefficients to better capture spatial dependence and heterogeneity? The answer to this question has important implications for whether the standard SAR model should be extended.

To test this hypothesis, we conducted a preliminary experiment to examine the variation in spatial correlation coefficients across different spatial units. Specifically, we defined a square region and divided it into a 15×15 grid, yielding 225 spatial units. In the experiment, the spatial correlation coefficient ρ for each unit was assigned a different value, following a sine function to simulate the heterogeneous spatial dependence that may exist across regions. The regression coefficients were fixed as $\beta =[1,2{]}^{⊤}$, and the explanatory variables *X* were generated using four different distributions: uniform distribution, normal distribution, chi-square distribution, and beta distribution. Specifically, for the uniform distribution, *X* was uniformly distributed within the interval [0,1]; for the normal distribution, *X* was drawn from a normal distribution with a mean of 0 and variance of 1; for the chi-square distribution, *X* was sampled from a chi-square distribution with 2 degrees of freedom; and for the beta distribution, *X* was generated using shape parameters α=2 and β=2. The error term ε was drawn from a normal distribution with a mean of 0 and a variance of 1. To ensure the robustness of the results, the experiment was repeated 300 times using a Monte Carlo simulation. Table 1 summarizes the results from applying the SAR model across 300 simulations for each distribution.

**Table 1:** SAR model estimation results for different distributions.

Distribution	Parameter	True Value	Estimated Value	S.E.	Bias	MSE
Uniform	ρ	- -	0.9534	- -	- -	- -
	$\beta_{1}$	1.0000	–0.9150	0.2357	–1.9150	3.6498
	$\beta_{2}$	2.0000	4.4748	0.4139	2.4748	7.9442
	$\sigma^{2}$	1.0000	38.5281	3.2456	37.5281	106.3059
Normal	ρ	- -	0.0678	- -	- -	- -
	$\beta_{1}$	1.0000	1.2478	0.1888	0.2478	0.0970
	$\beta_{2}$	2.0000	2.4565	0.2042	0.4565	0.2501
	$\sigma^{2}$	1.0000	6.0208	1.9522	5.0208	29.0190
Chi-Square	ρ	- -	0.6858	- -	- -	- -
	$\beta_{1}$	1.0000	0.8798	0.2715	0.1202	0.0882
	$\beta_{2}$	2.0000	2.1133	0.3243	0.1133	0.1180
	$\sigma^{2}$	1.0000	25.6878	9.9851	24.6878	709.1907
Beta	ρ	- -	0.8803	- -	- -	- -
	$\beta_{1}$	1.0000	–0.1306	0.5038	1.1306	1.5319
	$\beta_{2}$	2.0000	1.1893	0.5832	0.8107	0.9973
	$\sigma^{2}$	1.0000	5.3291	1.7058	4.3291	21.6505

The estimation results in Table 1 clearly demonstrate that the SAR model is inadequate for capturing heterogeneous spatial dependence. Under the uniform distribution, the estimated spatial correlation coefficient $\hat{\rho }=0.9534$ significantly deviates from the true value, showing that the SAR model tends to overestimate ρ when spatial correlation varies across regions. Additionally, the regression coefficients $\hat{\beta_{1}}=-0.9150$ and $\hat{\beta_{2}}=4.4748$ are heavily biased, deviating by 191.5% and 123.7% from their true values, respectively. The model also overestimates the variance parameter $\hat{\sigma^{2}}=38.5281$ by more than 37 times. These biases are not unique to the uniform distribution. Under the normal distribution, $\hat{\rho }=0.0678$ is far from the true value, indicating a failure of the model to correctly capture spatial dependence. The estimates of $\beta_{1}$ and $\beta_{2}$ also show significant bias, with $\beta_{1}=1.2478$ and $\beta_{2}=2.4565$ deviating from the true values by 24.8% and 22.8%, respectively. The model also overestimates $\sigma^{2}=6.0208$. Under the chi-square distribution, the model performs slightly better, with $\hat{\rho }=0.6858$, but the estimates for $\beta_{1}$ and $\beta_{2}$ remain biased, and $\sigma^{2}$ are grossly overestimated at 25.6878. The best performance is seen with the beta distribution, where $\hat{\rho }=0.8803$ is closer to the true value, but still exhibits bias. Similarly, $\beta_{1}=-0.1306$ and $\beta_{2}=1.1893$ deviate significantly from their true values, and $\hat{\sigma^{2}}=5.3291$ is still overestimated. In all cases, the SAR model fails to accurately estimate the spatial correlation, regression coefficients, and variance, confirming its limitations when dealing with spatially heterogeneous data.

### Spatial single-index varying coefficient autoregressive model

In the SAR model, spatial autocorrelation is primarily governed by the spatial weight matrix *W* and the spatial autoregressive coefficient ρ. The matrix *W* reflects the adjacency relationships among geographic locations or spatial units and is typically constructed using an adjacency matrix, a distance matrix, or a standardized matrix based on specific rules. The spatial correlation coefficient ρ quantifies the strength of association among spatial units, with its value usually ranging between –1 and 1, corresponding to negative and positive spatial effects, respectively.

However, as demonstrated in the previous section, the assumption of a constant spatial autoregressive coefficient ρ imposes inherent limitations on the model. Specifically, it fails to capture spatial heterogeneity, where the degree of spatial dependence may vary across different spatial units. In practical applications, spatial interactions are often influenced by region-specific factors such as geographic features, socioeconomic conditions, and policy environments. As a result, imposing a globally constant ρ may oversimplify and misrepresent the true spatial structure. To better capture underlying spatial heterogeneity, the model should be extended to allow unit-specific spatial autoregressive coefficients.

Consider the following SSIVCAR model:

$$y_{i}=g(u_{i}^{⊤}\alpha )\sum_{j=1}^{n}w_{ij}y_{j}+x_{i}^{⊤}\beta +\varepsilon_{i},$$ (2)

where *y*_*i*_ denotes the response variable, $\alpha =(\alpha_{1},\alpha_{2},\cdots ,\alpha_{m}{)}^{⊤}$ and $\beta =(\beta_{1},\beta_{2},\cdots ,\beta_{d}{)}^{⊤}$ are parameters to be estimated. The element *w*_*ij*_ denotes the (*i*,*j*)th entry of the given n×n spatial adjacency matrix *W*, and $x_{i}=(x_{i1},x_{i2},\cdots ,x_{id}{)}^{⊤}$ represents the observed variables. The error term $\varepsilon_{i}$ is assumed to be independent and normally distributed with mean 0 and variance $\sigma^{2}$. The function g(·) is an unknown single-index function that captures spatial heterogeneity, where $u_{i}=(u_{i1},u_{i2},\cdots ,u_{im}{)}^{⊤}$ denotes the spatial location parameters for the *i*th observation. By combining the spatial adjacency matrix with the single-index function g(·), the model flexibly reflects the influence of spatial heterogeneity while capturing spatial autocorrelation.

Compared to the SAR model, the SSIVCAR model replaces the global spatial autoregressive coefficient ρ with a nonlinear function $g(u_{i}^{⊤}\alpha )$. Since $g(u_{i}^{⊤}\alpha )$ follows the structure of a single-index model, this formulation not only captures spatial heterogeneity but also mitigates the curse of dimensionality.

### Model estimation

For ease of estimation, the model can be rewritten in matrix form as follows:

Y=GWY+Xβ+ε, (3)

where $Y=(y_{1},y_{2},\cdots ,y_{n}{)}^{⊤}$, $X=(x_{1},x_{2},\cdots ,x_{n}{)}^{⊤}$, and


$$G=\text{diag}\{g(u_{1}^{⊤}\alpha ),g(u_{2}^{⊤}\alpha ),\cdots ,g(u_{n}^{⊤}\alpha )\},$$

with $\varepsilon =(\varepsilon_{1},\varepsilon_{2},\cdots ,\varepsilon_{n}{)}^{⊤}$. Here, diag{·} denotes the diagonal matrix whose diagonal elements are the entries inside the braces.

Assuming that the matrix *I*–*GW* is nonsingular, equation (3) can be rewritten as:

$$Y=(I-GW{)}^{-1}(X\beta +\varepsilon ),$$ (4)

which further implies:

$$WY=W(I-GW{)}^{-1}(X\beta +\varepsilon ).$$ (5)

Assuming that *X* is exogenous, we analyze the relationship between *WY* and ε. Specifically, we have:


$$𝔼[(WY{)}^{⊤}\varepsilon ]=𝔼[(W(I-GW{)}^{-1}X\beta {)}^{⊤}\varepsilon +(W(I-GW{)}^{-1}\varepsilon {)}^{⊤}\varepsilon ]$$

$$=𝔼[\varepsilon^{⊤}W(I-GW{)}^{-1}\varepsilon ]$$

$$=\sigma^{2}\;𝔼[\text{tr}\{W(I-GW{)}^{-1}\}],$$ (6)

where tr(·) denotes the trace of a matrix. Since $𝔼[\varepsilon^{⊤}W(I-GW{)}^{-1}\varepsilon ]\neq 0$, it follows that there is a correlation between *WY* and ε in the SSIVCAR model, leading to an endogeneity problem.

Therefore, the SSIVCAR model cannot be directly estimated using conventional methods; and requires the use of appropriate instrumental variables or alternative estimation techniques to address endogeneity.

The detailed estimation procedure is as follows:

**Step 1**: To estimate the single-index component $g(u_{i}^{⊤}\alpha )$, an initial value $\alpha_{0}$ is first specified. This initial estimate can be obtained using the de-nonlinearization method proposed by Xue *et al*. [36] to estimate α in the model Y=UαWY+Xβ+ε, which then serves as the initial value for the SSIVCAR model. Alternatively, when the dimension of *U* is low, a grid search method can be employed to obtain a more accurate initial estimate.

**Step 2**: Let $a\leq k_{1}<k_{2}<\cdots <k_{l}\leq b$ be *l* knots on the interval [*a*,*b*]. Given $\alpha_{0}$, define $t_{i}=u_{i}^{⊤}\alpha_{0}$, which is used to construct the basis functions. The *p*th-order truncated power spline basis function is given by:

$$B(t_{i})=(1,t_{i},t_{i}^{2},\cdots ,t_{i}^{p},(t_{i}-k_{1}{)}_{+}^{p},\cdots ,(t_{i}-k_{l}{)}_{+}^{p}{)}^{⊤},$$ (7)

where the notation $(t_{i}-k_{l}{)}_{+}^{p}$ is defined as:

$$(t_{i}-k_{l}{)}_{+}^{p}=\left\{ \begin{array}{cc} (t_{i}-k_{l}{)}^{p}, & \text{if }t_{i}>k_{l},\\ 0, & \text{if }t_{i}\leq k_{l}. \end{array}\right.$$ (8)

By constructing the spline basis function *B*(*t*_*i*_), we can flexibly approximate the unknown function *g*(*t*). In subsequent iterations, *t*_*i*_ is updated based on the current value of α, thereby optimizing the estimation of the model parameters. Let the coefficients for the truncated power spline basis functions be $\delta =(\delta_{0},\delta_{1},\cdots ,\delta_{l+p+1}{)}^{⊤}$. Then,

$$g(t_{i})\approx B(t_{i}{)}^{⊤}\delta .$$ (9)

Substituting this approximation into equation (3) yields:

$$Y=\tilde{G}WY+X\beta +\varepsilon ,$$ (10)

where $\tilde{G}=\text{diag}\{B(t_{1}{)}^{⊤}\delta ,B(t_{2}{)}^{⊤}\delta ,\cdots ,B(t_{n}{)}^{⊤}\delta \}$ and


$$B=(\begin{array}{c} B(t_{1}{)}^{⊤}\\ B(t_{2}{)}^{⊤}\\ \vdots \\ B(t_{n}{)}^{⊤} \end{array}).$$

As mentioned earlier, since the model suffers from an endogeneity problem and *X* is exogenous, one can choose linearly independent variables including *X*, *WX*, ${\text{W}}^{2}\text{X}$, *WU*, and ${\text{W}}^{2}\text{U}$ as instrumental variables for *WY*.

**Step 3**: Let *Q* be the matrix of instrumental variables constructed from the linearly independent variables *X*, *WX*, ${\text{W}}^{2}\text{X}$, *WU*, and ${\text{W}}^{2}\text{U}$. Then, the two-stage least squares (2SLS) method can be used for estimation.

In the first stage, to address the endogeneity problem, the fitted values of *WY* are estimated as:

$$\hat{WY}=Q(Q^{⊤}Q{)}^{-1}Q^{⊤}WY.$$ (11)

Substituting $\hat{WY}$ into equation (10) transforms the model into:

$$Y=\tilde{G}\;Q(Q^{⊤}Q{)}^{-1}Q^{⊤}WY+X\beta +\varepsilon .$$ (12)

At this point, the endogeneity problem has been addressed through the instrumental variable matrix *Q*.

In the second stage, the parameters β and δ are estimated based on the modified model. Specifically, we have:

$$\hat{\beta }=(X^{⊤}X{)}^{-1}X^{⊤}(I-\tilde{G}\;Q(Q^{⊤}Q{)}^{-1}Q^{⊤}W)Y,$$ (13)

$$\hat{\delta }=(B^{⊤}S^{⊤}SB{)}^{-1}B^{⊤}S^{⊤}(Y-X\hat{\beta }),$$ (14)

where $S=\text{diag}(Q(Q^{⊤}Q{)}^{-1}Q^{⊤}WY)$.

This two-stage estimation procedure addresses the endogeneity problem while ensuring the efficiency and consistency of the parameter estimates.

**Step 4**: To ensure model identifiability, we impose a parameter constraint using the “omit-one-component" method. Specifically, let $\phi =(\alpha_{2},\alpha_{3},\cdots ,\alpha_{m}{)}^{⊤}$. Then, we reparameterize α as:

$$\alpha (\phi )=(\begin{array}{c} \sqrt{1-\|\phi {\|}^{2}}\\ \phi \end{array}).$$ (15)

Substituting the parameter estimates $\hat{\beta }$ and $\hat{\delta }$ obtained in Step 3 into equation (3), we construct the loss function:

$$L=\|Y-(\tilde{G}(\alpha )\hat{WY}+X\hat{\beta }){\|}_{2}^{2},$$ (16)

where $\tilde{G}(\alpha )=\text{diag}\{B(\alpha )\delta \}$. We then minimize the loss function *L* using the Trust Region Method (TRM) [37] to obtain an estimate $\hat{\phi }$, from which a new estimate of α, denoted by $\hat{\alpha }$, is computed.

**Step 5**: Substitute the estimate $\hat{\alpha }$ obtained in Step 4 back into Step 2, and then repeat Steps 2 through 4 until $\hat{\alpha }$ converges. The final converged value is the optimal estimate ${\hat{\alpha }}_{opt}$.

**Step 6**: Substitute the optimal estimate ${\hat{\alpha }}_{opt}$ from Step 5 into Steps 2 through 4 to obtain the optimal estimates of the model parameters: ${\hat{\beta }}_{opt}$, ${\hat{\delta }}_{opt}$, and ${\hat{\sigma }}_{opt}^{2}$. The predicted values of the model, $\hat{Y}$, are given by:

$$\hat{Y}=\tilde{G}({\hat{\alpha }}_{opt})\hat{WY}+X{\hat{\beta }}_{opt},$$ (17)

and the optimal estimate of the residual variance is:

$${\hat{\sigma }}_{opt}^{2}=\frac{1}{n}(Y-\hat{Y}{)}^{⊤}(Y-\hat{Y}).$$ (18)

In summary, through the iterative optimization process described above, we obtain the optimal estimates of the model parameters α, β, δ, and $\sigma^{2}$.

## Monte Carlo simulation

This chapter conducts a Monte Carlo simulation study to evaluate the performance of the spatial lag single-index varying coefficient model under finite sample conditions. Simulations were performed on a computer running 64-bit Windows 11 with a 2.2GHz AMD processor, using MATLAB 2024b as the programming environment. Optimization was carried out using MATLAB’s Trust-Region Method (TRM).

### Evaluation metrics

To assess the accuracy of the parameter estimates, we employ the following three statistical measures:

- **Standard Error (S.E.)**:
  $$S.E.=\sqrt{\frac{1}{n_{\text{mc}}}\sum_{i=1}^{n_{\text{mc}}}({\hat{\theta }}_{i}-\bar{\hat{\theta }}{)}^{2}},$$ (19)
- where ${\hat{\theta }}_{i}$ denotes the estimated value of the parameter θ in the *i*th simulation and $\bar{\hat{\theta }}$ is the average estimate.
- **Bias**:
  $$Bias=|\bar{\hat{\theta }}-\theta |,$$ (20)
- where θ is the true value of the parameter.
- **Mean Squared Error (MSE)**:
  $$MSE=\frac{1}{n_{\text{mc}}}\sum_{i=1}^{n_{\text{mc}}}({\hat{\theta }}_{i}-\theta {)}^{2},$$ (21)
- where $n_{\text{mc}}$ represents the total number of simulations.

Lower values of these measures indicate greater estimation accuracy and a better approximation of the unknown function.

### Construction of spatial location information and model specification

**Fig 1:**
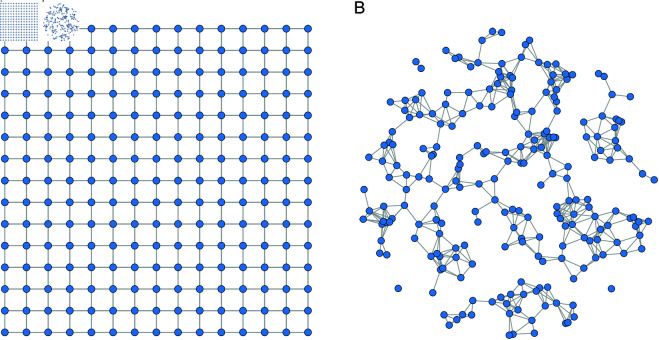
Illustration of spatial unit configurations: (a) regular square grid, (b) randomly scattered points within a circle with k-nearest neighbor connections.

In the simulation experiments, two types of spatial region structures are considered to reflect different forms of spatial organization: (1) a regular square grid with evenly spaced locations, and (2) an irregular circular region with randomly distributed points. Schematic illustrations of these two spatial settings are shown in Fig 1.

(1) **Regular square grid setting:** Assume that *m* = 2, so that geographic locations are represented by two-dimensional coordinates. The simulation region is a square, with its lower-left corner designated as the origin. A Cartesian coordinate system is imposed along the horizontal and vertical directions. Each side of the square is divided into *h*–1 equal segments. By connecting the division points along both axes, a total of h×h intersection points are generated, including those on the boundary. These intersection points represent the simulated geographic locations.

Assuming a sample size of *n* = *h*^2^, the coordinates of each geographic location $U_{i}=(u_{i1},u_{i2}{)}^{⊤}$ can be defined as:

$$u_{i}=(\begin{array}{c} 0.5\cdot ((i-1)\mathrm{mod}h)\\ 0.5\cdot \left\lfloor \frac{i-1}{h}\right\rfloor \end{array}),$$ (22)

where (i−1)modh denotes the remainder when *i*–1 is divided by *h*, and $\left\lfloor \frac{i-1}{h}\right\rfloor$ denotes the integer part of the quotient; where, ⌊·⌋ denotes the floor function.

(2) **Irregular circular region setting:** To better reflect the fact that real-world spatial structures are often irregular and do not conform to regular grids, we introduce an additional simulation setting based on a circular region with randomly distributed spatial units and irregular neighborhood structures. Specifically,*n* spatial units are generated by randomly placing *n* points within a unit circle. Each spatial location $U_{i}=(u_{i1},u_{i2}{)}^{⊤}$ is drawn from a uniform distribution over the disk $\left\{(x,y):x^{2}\;+\;y^{2}\leq R\right\}$. To construct the spatial weight matrix *W*, we adopt a *k*-nearest neighbor strategy: for each point *U*_*i*_, we identify its *k* closest neighbors (measured by Euclidean distance) and assign a weight of 1 to each of these links, setting all other weights to 0. The resulting adjacency matrix is then row-normalized.

**Model specification:** The SSIVCAR model is specified as follows:

$$y_{i}=g(u_{i}^{⊤}\alpha )\sum_{j=1}^{n}w_{ij}y_{j}+x_{i}^{⊤}\beta +\varepsilon ,$$ (23)

where the sample size is set to *n* = 225, with parameters $\alpha =(\frac{1}{2},\frac{\sqrt{3}}{2}{)}^{⊤}$ and $\beta =(1,2{)}^{⊤}$. The covariates *x*_*ij*_ are independently drawn from the uniform distribution *U*(–1,1),with *h* = 15, and *u*_*i*_ is as defined in (22). The elements *w*_*ij*_ are drawn from the Rook contiguity matrix *W* (in the square grid case) or from the *k*-nearest neighbor matrix (in the circular region). The coefficient function is specified as g(t)=sin(t), and the error term ε is independently drawn from the normal distribution $\text{N}(0,1^{2})$.

### Comparative experiments

Parameter estimation is conducted under the following settings. The number of simulations is set to $n_{\text{mc}}=300$, following standard practice in Monte Carlo studies. A key principle of Monte Carlo simulations is that increasing the number of simulations generally leads to more stable and accurate estimates, particularly for complex models. The choice of 300 simulations ensures sufficient convergence of the results without excessive computational cost. The node step is set to 10, motivated by the use of spline functions to approximate the unknown function *g*. The choice of step size in spline-based methods directly affects the resolution of the approximation. Specifically, it determines how frequently the index variable is sampled, thereby affecting the spline’s ability to capture the underlying behavior of the function. A smaller step size yields a finer discretization and more accurate approximation of *g*, but increases computational burden. Conversely, a larger step would decrease the model’s ability to accurately capture variations in the function. In this study, to select the nodes, we sort the independent variable of the unknown function and choose every 10th value as a node. This method ensures that the nodes are spaced evenly, providing a well-distributed sample of the independent variable. The order of the spline basis functions is set to p=3. The choice of *p* is driven by the need for a balance between model flexibility and smoothness. In spline approximation, the order *p* controls the degree of smoothness and the model’s ability to fit the data. A higher order increases the flexibility of the splineand may lead to overfitting, whereas a lower order may fail to adequately capture the complexity of the underlying function. Setting *p* = 3 is a common choice in spline-based approximation, as it provides sufficient flexibility to approximate complex functions while maintaining smoothness and avoiding overfitting.

Table 2 presents the average estimated values, S.E., biases, and mean MSE for the various parameters.

**Table 2:** Statistics of parameter estimation results under different spatial structures.

Structure	Parameter	True Value	Estimated Value	S.E.	Bias	MSE
Regular square	$\alpha_{1}$	0.5000	0.5082	0.0454	0.0082	0.0021
	$\alpha_{2}$	0.8660	0.8596	0.0271	0.0064	0.0008
	$\beta_{1}$	1.0000	1.0158	0.2704	0.0158	0.0733
	$\beta_{2}$	2.0000	1.9707	0.2414	0.0293	0.0591
	$\sigma^{2}$	1.0000	1.0868	0.3045	0.0868	0.1003
Irregular circular	$\alpha_{1}$	0.5000	0.4955	0.1331	0.0045	0.0177
	$\alpha_{2}$	0.8660	0.8532	0.0935	0.0128	0.0089
	$\beta_{1}$	1.0000	0.9004	0.1669	0.0996	0.0378
	$\beta_{2}$	2.0000	1.8673	0.2077	0.1327	0.0608
	$\sigma^{2}$	1.0000	1.1800	0.4123	0.1800	0.2024

Table 2 shows the parameter estimation results for a sample size of *n* = 225 and *n*_*mc*_ = 300 simulations. The results indicate that the model exhibits good parameter estimation precision and stability under small sample conditions. The estimates for the single-index parameters $\alpha_{1}$ and $\alpha_{2}$ show small biases and low values of both MSE and S.E., indicating a high accuracy in capturing spatial location effects. In addition, the regression parameters $\beta_{1}$ and $\beta_{2}$ are close to their true values, with biases within an acceptable range, although the MSE is slightly higher, as expected under a finite sample. It is worth noting that the standard error for $\beta_{1}$ is relatively large, suggesting some uncertainty in its estimation under small-sample conditions. The estimate of the error variance $\sigma^{2}$ is close to the true value, though its standard error is relatively high, possibly due to the influence of random variation. Overall, the estimation results under small-sample conditions demonstrate the model’s robustness and adaptability. The estimation precision for the single-index parameters is particularly notable, while the regression parameters and error variance remain within reasonable bounds.

**Fig 2:**
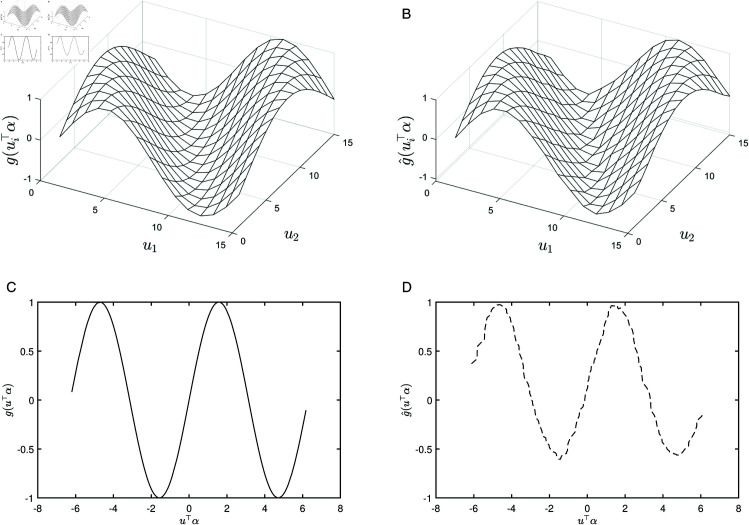
Comparison of the true and fitted data under different spatial regions. (**a**) True surface for square region; (**b**) fitted surface for square region; (**c**) true curve for circular region; (**d**) fitted curve for circular region.

Furthermore, the fitting performance of the function *g* is illustrated in Fig 2, where Fig 2(a) shows the true surface for the square region and Fig 2(b) shows the fitted surface for the square region. Fig 2(c) shows the true curve and Fig 2(d) presents the fitted curve for the circular region. These plots enable a direct comparison of the fitting accuracy across regular (square) and irregular (circular) spatial domains.

Fig 2 compares the true and fitted representations of the unknown function *g* across two distinct spatial regions. The true surface for the square region (Fig 2 (a)) exhibits smooth variation with respect to the spatial location parameter $u_{i}^{⊤}\alpha$, showing periodic fluctuations. The fitted surface for the square region (Fig 2 (b)) successfully reproduces these global features, particularly the amplitude and trends near the extrema, confirming the model’s ability to capture the main characteristics of the underlying function. For the circular region, the true curve (Fig 2 (c)) presents a non-periodic pattern reflecting the more irregular distribution of the spatial locations. The fitted curve (Fig 2 (d)) closely aligns with the true curve, indicating that the model can effectively accommodate irregular spatial structures. Although some minor discrepancies are observed in finer details, the overall fit remains strong, suggesting that the spatial lag single-index varying coefficient model is robust for both regular and irregular spatial structures.

To further evaluate the distributional characteristics and normality of the parameter estimates, we present histograms and Q-Q plots for the parameters $\alpha_{1}$, $\alpha_{2}$, $\beta_{1}$, and $\beta_{2}$. The histograms provide an intuitive depiction of the distributional concentration of the estimates, while the Q-Q plots verify whether the estimates conform to the normality assumption.

**Fig 3:**
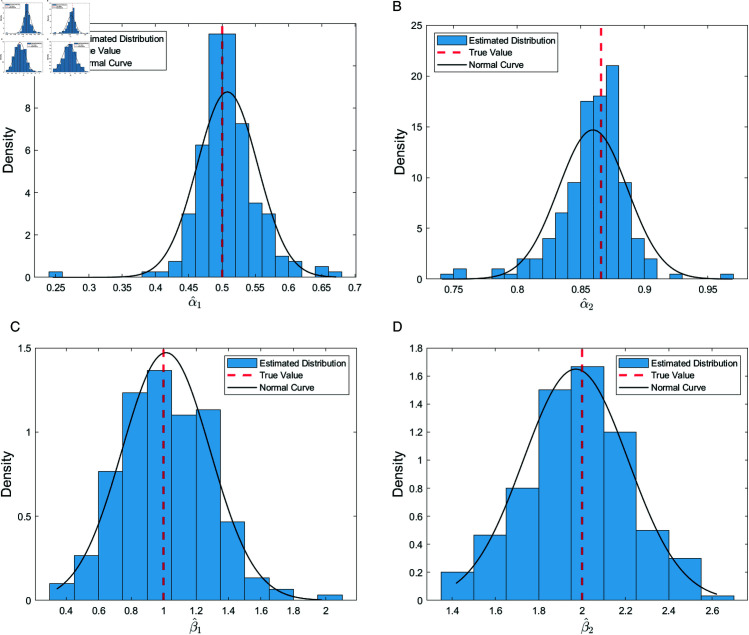
Histograms of the parameter estimates. (**a**) Distribution of $\alpha_{1}$; (**b**) distribution of $\alpha_{2}$; (**c**) distribution of $\beta_{1}$; (**d**) distribution of $\beta_{2}$.

**Fig 4:**
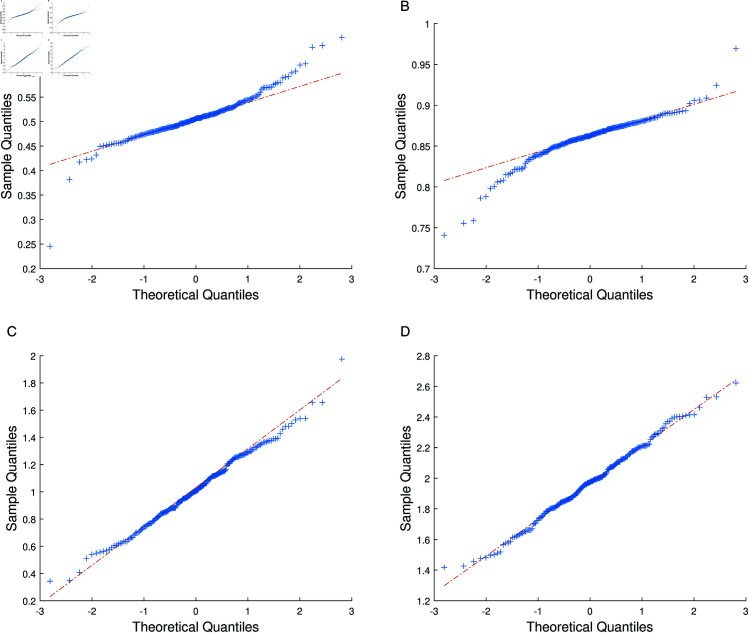
Q-Q plots of the parameter estimates. (**a**) Q-Q plot for $\alpha_{1}$; (**b**) Q-Q plot for $\alpha_{2}$; (**c**) Q-Q plot for $\beta_{1}$; (**d**) Q-Q plot for $\beta_{2}$.

Fig 3 and 4 demonstrate that the estimates for $\alpha_{1}$, $\alpha_{2}$, $\beta_{1}$, and $\beta_{2}$ are well concentrated and approximately normally distributed. The histograms reveal that the estimates of $\alpha_{1}$ (Fig 3 (a)) and $\alpha_{2}$ (Fig 3 (b)) exhibit symmetric, bell-shaped distributions centered around the true values, indicating high precision and stability in estimating the single-index parameters. For $\beta_{1}$ (Fig 3 (c)) and $\beta_{2}$ (Fig 3 (d)), although the tails are slightly heavier, the overall distribution is consistent with normality. The corresponding Q-Q plots (Fig 4) further confirm that most points align closely with the theoretical diagonal, with only minor deviations in the tails, thereby validating the normality assumption of the parameter estimates.

In the following analysis, the regular square spatial structure is used as the baseline to examine how different values of $\sigma^{2}$ and alternative specifications of spatial adjacency matrices influence the model’s estimation performance.

To further evaluate the model’s performance, we compared the parameter estimation results under different error variances $\sigma^{2}$ (i.e., $\sigma^{2}=0.64$, 0.25, 0.01). Table 3 presents the estimated parameter values, S.E., Biases, and MSE under these three conditions. This comparison facilitates analysis of how the estimation accuracy and stability vary with different levels of error variance.

**Table 3:** Parameter estimation results under different $\sigma^{2}$ conditions.

Parameter	$\sigma^{2}=0.64$				$\sigma^{2}=0.25$				$\sigma^{2}=0.01$			
	Estimate	S.E.	Bias	MSE	Estimate	S.E.	Bias	MSE	Estimate	S.E.	Bias	MSE
$\alpha_{1}$	0.5064	0.0351	0.0064	0.0013	0.4990	0.0241	0.0010	0.0006	0.4999	0.0026	0.0001	0.0000
$\alpha_{2}$	0.8613	0.0210	0.0047	0.0005	0.8661	0.0158	0.0001	0.0003	0.8661	0.0015	0.0000	0.0000
$\beta_{1}$	0.9996	0.2229	0.0004	0.0497	1.0022	0.1555	0.0022	0.0242	1.0053	0.0837	0.0053	0.0070
$\beta_{2}$	1.9877	0.2154	0.0123	0.0466	1.9659	0.1464	0.0341	0.0226	1.9681	0.0786	0.0319	0.0072
$\sigma^{2}$	0.6736	0.1658	0.0336	0.0435	0.2442	0.0844	0.0058	0.0725	0.0104	0.0020	0.0004	0.0080

As shown in Table 3, as $\sigma^{2}$ decreases, the estimation accuracy of the model parameters improves notably as $\sigma^{2}$ decreases: the estimates approach the true values, and both S.E. and MSE decrease substantially. For the single-index parameters $\alpha_{1}$ and $\alpha_{2}$, both bias and MSE are relatively large under $\sigma^{2}=0.64$, but they decrease rapidly as $\sigma^{2}$ is reduced to 0.25 and 0.01, with the MSE nearly approaching zero. Similarly, the regression parameters $\beta_{1}$ and $\beta_{2}$ exhibit a comparable pattern: both standard errors and MSE are large under $\sigma^{2}=0.64$ due to high noise, but decrease substantially as $\sigma^{2}$ becomes smaller. For the error variance $\sigma^{2}$ itself, the estimates converge to the true value and exhibit lower S.E. and MSE as the true variance decreases. These results further validate the model’s adaptability and robustness under varying levels of error variance.

Thus, the comparative experiments indicate that lower error variance leads to improved estimation accuracy, with estimates being closer to the true values and exhibiting significantly lower standard errors and MSE.

**Fig 5:**
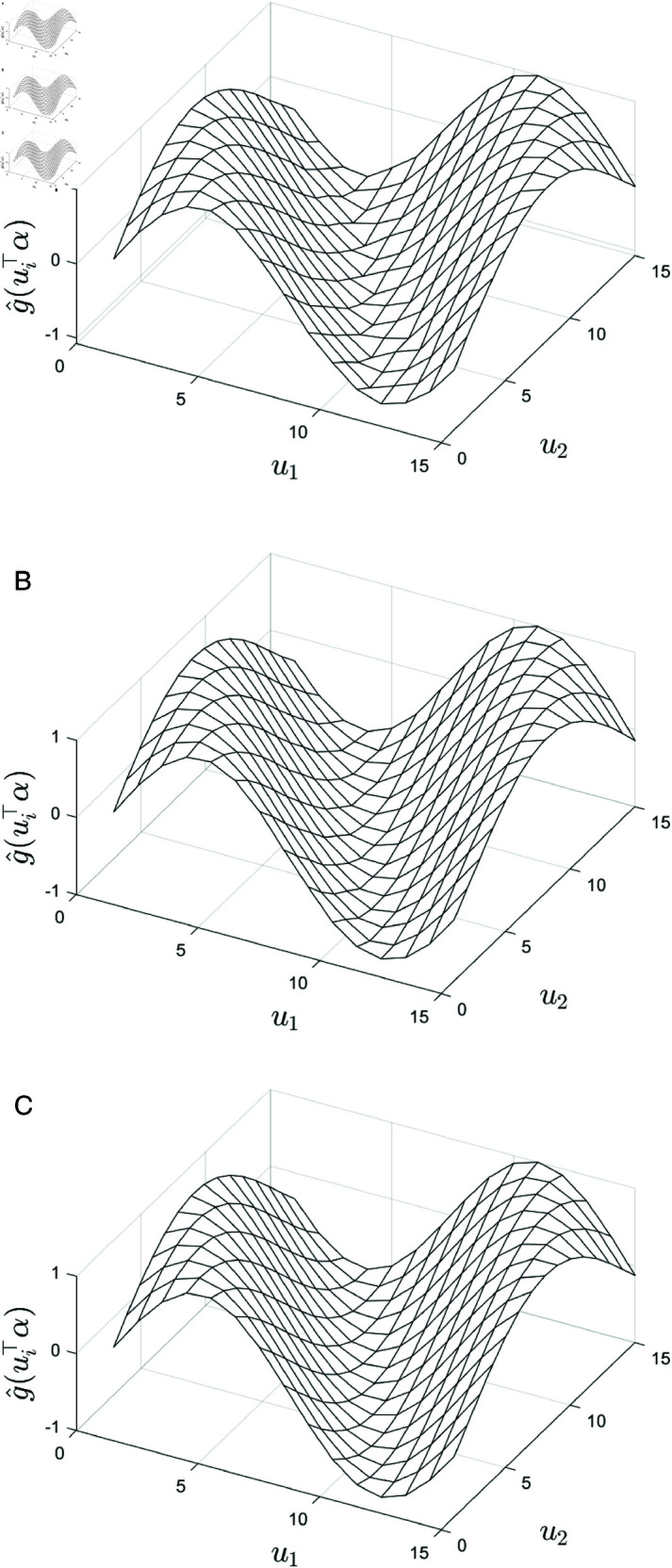
Fitting results of the unknown function g under different $\sigma^{2}$ conditions. (**a**) $\sigma^{2}=0.64$; (**b**) $\sigma^{2}=0.25$; (**c**) $\sigma^{2}=0.01$.

Fig 5 illustrates the fitted results for the unknown function *g* under different error variances $\sigma^{2}$ ($\sigma^{2}=0.64$, 0.25, 0.01).

Fig 5 shows that as $\sigma^{2}$ decreases, the fitted performance of the unknown function *g* improves substantially. When $\sigma^{2}=0.64$ (Fig 5 (a)), the fitted surface captures the overall trend but displays noticeable local discrepancies; As $\sigma^{2}$ decreases to 0.25 (Fig 5 (b)), the differences between the fitted and true surfaces diminish, enabling the model to more accurately capture the global structure of the function; and when $\sigma^{2}=0.01$ (Fig 5 (c)), the fit is nearly perfect, with the fitted surface almost exactly matching the true surface.

Fig 5 presents the fitted results for the unknown function *g* under three distinct noise variance settings. As the variance $\sigma^{2}$ decreases, the fitting performance improves progressively. When $\sigma^{2}=0.64$, the model captures the global shape of the function but exhibits noticeable deviations in regions of high curvature. For $\sigma^{2}=0.25$, the estimation becomes more stable, with reduced deviations in both peak and valley regions. At a low noise level of $\sigma^{2}=0.01$, the fitted surface shows excellent agreement with the true function, confirming the model’s robustness under low-noise conditions. These results demonstrate that lower noise variance leads to improved estimation accuracy, particularly in regions where the function exhibits rapid variation.

To further analyze the impact of spatial adjacency structures on parameter estimation, we consider four types of spatial weight matrices: the Bishop matrix, the Case matrix, the Distance matrix, and a random matrix. For each matrix, we recorded the estimated values, S.E., Biases, and MSE of the parameters. The Bishop and Case matrices define weights based on neighborhood structures and are suited to capturing local and complex spatial dependencies, respectively; the Distance matrix characterizes global spatial dependence based on pairwise distances; while the random matrix, independent of geographic location, allocates weights based on other associated features, offering broader applicability. Table 4 presents the parameter estimation results under each of the four spatial weight matrix settings.

**Table 4:** Parameter estimation results under different spatial adjacency matrices.

Bishop					Case				
Parameter	Estimate	S.E.	Bias	MSE	Parameter	Estimate	S.E.	Bias	MSE
$\alpha_{1}$	0.5007	0.0543	0.0007	0.0030	$\alpha_{1}$	0.5380	0.1085	0.0380	0.0132
$\alpha_{2}$	0.8633	0.0326	0.0028	0.0011	$\alpha_{2}$	0.8327	0.0732	0.0333	0.0065
$\beta_{1}$	0.9882	0.2339	0.0118	0.0548	$\beta_{1}$	0.9841	0.3038	0.0159	0.0925
$\beta_{2}$	1.9968	0.2424	0.0032	0.0588	$\beta_{2}$	1.9785	0.2996	0.0215	0.0902
$\sigma^{2}$	1.0585	0.2359	0.0585	0.0591	$\sigma^{2}$	0.9213	0.1145	0.0787	0.0301
**Distance**					**Random Matrix**				
**Parameter**	**Estimate**	**S.E.**	**Bias**	**MSE**	**Parameter**	**Estimate**	**S.E.**	**Bias**	**MSE**
$\alpha_{1}$	0.5021	0.0382	0.0021	0.0015	$\alpha_{1}$	0.5171	0.0678	0.0171	0.0049
$\alpha_{2}$	0.8637	0.0223	0.0024	0.0005	$\alpha_{2}$	0.8522	0.0418	0.0138	0.0019
$\beta_{1}$	0.9613	0.2599	0.0387	0.0691	$\beta_{1}$	1.0074	0.2753	0.0074	0.0758
$\beta_{2}$	1.9571	0.2349	0.0429	0.0570	$\beta_{2}$	1.9567	0.2609	0.0433	0.0699
$\sigma^{2}$	0.8576	0.0872	0.1424	0.0279	$\sigma^{2}$	0.9402	0.1298	0.0598	0.0204

As shown in Table 4, the parameter estimation results vary under different spatial adjacency matrices. The Bishop matrix performs exceptionally well in capturing local spatial relationships, with very small biases and low MSE for $\alpha_{1}$ and $\alpha_{2}$, indicating that it effectively characterizes local dependency. The Case matrix demonstrates high flexibility in scenarios with strong spatial dependence, producing estimates for $\beta_{1}$ and $\beta_{2}$ that are close to their true values and stable estimation of $\sigma^{2}$. The Distance matrix shows remarkable advantages in modeling global spatial relationships, achieving the highest estimation accuracy for $\alpha_{1}$ and $\alpha_{2}$ , along with highly stable estimates for $\beta_{1}$ and $\beta_{2}$. The random matrix exhibits a balanced performance, with biases and MSE for all parameters within a reasonable range, indicating its adaptability in capturing both global and local relationships. These findings confirm that the choice of spatial weight matrix significantly affects model performance. The Bishop and Distance matrices offer distinct advantages for local and global modeling, respectively, while the Case and random matrices provide flexible alternatives for capturing complex spatial dependencies, offering diverse options for practical applications.

**Fig 6:**
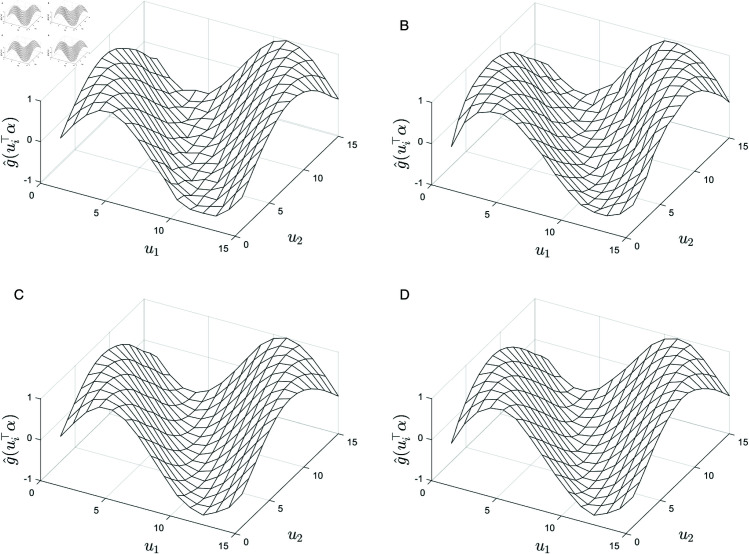
Fitting results of the unknown function g under different adjacency matrices. (**a**) Bishop matrix; (**b**) case matrix; (**c**) distance matrix; (**d**) random matrix.

Fig 6 presents the fitted results for the unknown function *g* under four spatial weight matrices: Bishop, Case, Distance, and Random. The figure clearly illustrates how different spatial weight matrices influence the fitting performance.

Fig 6 presents the estimated surfaces of the coefficient-modulating function $g(u_{i}^{⊤}\alpha )$ under four spatial weight matrices: Bishop, Case, Distance, and Random. While the overall shape of the estimated surfaces remains consistent—reflecting the smooth sinusoidal structure of the true function—subtle differences among them highlight the influence of the spatial weight matrix on estimation precision. Specifically, the Bishop and Case matrices, which emphasize local connectivity, result in slightly sharper surface transitions, particularly along the diagonal regions where spatial dependence is strongest. The Distance matrix yields a smoother and more stable surface, suggesting its strength in capturing global spatial patterns. In contrast, the Random matrix, although not based on geographic proximity, still produces a reasonably smooth fit, indicating its ability to balance local and global spatial information in a data-driven manner.

## Empirical analysis: The impact of digital economy development on the environment

In recent years, the digital economy has emerged as a key driver of global economic transformation. It not only promotes technological progress, optimizes resource allocation, and enhances production efficiency, but also offers new pathways for addressing environmental challenges. Through low-carbon technological innovation, increased informatization, and improved energy efficiency, the digital economy is expected to enhance environmental quality alongside economic growth. However, the actual impact of the digital economy on environmental governance remains uncertain. On one hand, it may reduce resource waste and optimize energy structures, thereby benefiting the environment; on the other hand, its expansion may result in higher energy consumption and carbon emissions, with its environmental effects exhibiting significant spatial heterogeneity across urban areas.

Against this background, this section uses Chinese cities as the study sample to examine the impact of digital economy development on environmental quality. The analysis addresses the following key questions: (1) Does digital economy development significantly improve environmental quality? (2) Does this impact vary spatially across regions with different geographic and economic characteristics? (3) When accounting for spatial effects, what mechanisms underlie the influence of digital economy development on various environmental indicators?In addition, the empirical analysis integrates urban environmental data with digital economy indicators to explore their spatial interrelationship, thereby offering new insights into the coordinated development of the digital economy and environmental governance.

All variables used in this study are derived from data for the year 2022, which is the most recent year with comprehensive and consistent records across all Chinese prefecture-level cities. Although the original data sources cover multiple years, this study adopts a cross-sectional design to focus on spatial heterogeneity rather than temporal variation. Potential dynamic effects of digital economy development on environmental quality will be examined in future research using panel data models.

To comprehensively examine the impact of digital economy development on urban environmental quality in China, this study defines environmental quality as the condition of the urban natural environment, as reflected by levels of air and industrial pollution. This definition is consistent with prior research in environmental economics and urban studies, where environmental quality is typically measured by pollution indicators that reflect environmental pressure and degradation caused by anthropogenic activities.

Based on this definition, four indicators are selected to characterize environmental quality, covering two primary dimensions: air pollution and industrial pollution. The selected indicators include average PM2.5 concentration, industrial wastewater discharge, industrial smoke and dust emissions, and industrial sulfur dioxide emissions. The average PM2.5 concentration (unit: $\mu g/m^{3}$) reflects the level of fine particulate matter in ambient air and serves as a key indicator of urban air pollution. Industrial wastewater discharge (unit: ten thousand tons) captures the intensity of water pollution resulting from industrial processes. Industrial smoke and dust emissions (unit: ten thousand tons) and industrial sulfur dioxide emissions (unit: ten thousand tons) quantify the release of solid particles and acidic gases from industrial operations, respectively. All data are sourced from the *China Urban Statistical Yearbook*. These indicators offer a comprehensive and objective representation of urban environmental quality.

In addition to environmental quality outcomes, the empirical model includes explanatory variables capturing the level of digital economy development and other socioeconomic characteristics.

The digital economy indicator serves as the primary explanatory variable in the model. To capture the multidimensional characteristics of urban digitalization, a composite index is constructed using the entropy method based on five components: the Digital Inclusive Finance Index (sourced from the *Peking University Digital Finance Research Center*), the number of international internet users per 100 people, the proportion of employees in the information transmission, computer service, and software industries, per capita telecommunication service volume, and the number of mobile phone subscribers per 100 people. The latter four indicators are sourced from the *China Urban Statistical Yearbook* [44].

To account for other factors potentially affecting environmental quality, five control variables are included in the model. These include: (1) population density, capturing the intensity of population concentration and its associated environmental burden; (2) per capita GDP, indicating the level of regional economic development; (3) foreign direct investment, measured as the ratio of actual utilized foreign capital to GDP, reflecting the degree of economic openness; (4) scientific expenditure, defined as the proportion of fiscal expenditure allocated to science and technology, reflecting government support for innovation; and (5) green innovation, measured by the number of authorized green patents, indicating the technological capacity for environmentally sustainable development. Among these variables, scientific expenditure and green innovation are also interpreted as partial indicators of environmental governance capacity, understood as institutional and policy mechanisms for managing environmental outcomes.

Taken together, the selected variables span multiple dimensions—including environmental quality, digital economy, and governance capacity—and thus provide a comprehensive foundation for analyzing the mechanisms through which digital economy development affects urban environmental outcomes. All variables used in this study are derived from data for the year 2022, which is the most recent year with comprehensive and consistent records across all Chinese prefecture-level cities. Although the original data sources span multiple years, this study adopts a cross-sectional design to focus on spatial heterogeneity rather than temporal variation. The potential dynamic effects of digital economy development on environmental quality will be examined in future research using panel data models. Table 5 summarizes the definitions and data sources of all variables used in the empirical analysis.

**Table 5:** Description and sources of main variables.

Type	Variable	Description	Source
Response Variables	Average PM2.5	Measured in $\mu g/m^{3}$, reflects urban air quality	China Urban Statistical Yearbook
	Industrial Wastewater Discharge	Measured in ten thousand tons; indicates the impact of industrial production on water pollution	China Urban Statistical Yearbook
	Industrial Smoke and Dust Emissions	Measured in ten thousand tons; represents particulate emissions during industrial production	China Urban Statistical Yearbook
	Industrial SO_2_ Emissions	Measured in ten thousand tons; assesses the impact of industrial emissions on the atmospheric environment	China Urban Statistical Yearbook
Explanatory Variables	Digital Economy Indicator	Composite score computed using the entropy method	Peking University Digital Finance Research Center
	Population Density	Number of permanent residents per unit area, reflecting urban population concentration	China Urban Statistical Yearbook
	Per Capita GDP	Measured in ten thousand yuan; indicates the level of economic development	China Urban Statistical Yearbook
	Foreign Direct Investment	Ratio of actual utilized foreign capital to GDP, reflecting the degree of economic openness	China Urban Statistical Yearbook
	Scientific Expenditure	Ratio of technology spending to fiscal expenditure, indicating technological investment	China Urban Statistical Yearbook
	Green Innovation	Number of green patent authorizations, reflecting green technology innovation capability	China Urban Statistical Yearbook

During data preprocessing, all variables were transformed using the natural logarithm to reduce scale differences and improve distributional smoothness. Table 6 presents the descriptive statistics of the main variables in logarithmic form, including the mean, standard deviation, median, minimum, maximum, skewness, and kurtosis.

**Table 6:** Descriptive statistics of log-transformed variables.

Variable	Mean	Std. Dev.	Median	Min	Max	Skewness	Kurtosis
lnDE	-5.6424	0.3856	-5.7070	-7.0881	-4.0106	1.2690	6.5496
lnPD	5.7450	0.9291	5.8944	1.7918	7.8548	-0.8895	4.3320
lnGDPPC	11.0690	0.4618	11.0140	9.8992	12.2930	0.3134	2.5623
lnFDI	-7.2030	1.7218	-7.1091	-13.5450	-4.3819	-0.7464	3.3745
lnSCI	-4.4368	1.0929	-4.3179	-7.0163	-1.9501	-0.2016	2.2492
lnGI	5.5736	1.7740	5.4424	-11.5130	9.8113	-2.7312	30.8390
lnPM2.5	3.3349	0.3659	3.3549	0.7955	3.9340	-2.8132	19.2900
lnIWW	7.6698	1.3553	7.8091	1.0986	11.1220	-1.0360	5.9421
lnIDP	8.4377	1.1919	8.4737	3.4657	12.3530	-0.2524	4.6804
$\ln ISO_{2}$	8.3280	1.0292	8.3436	4.3820	10.6940	-0.6101	4.1935

Table 6 shows that the variables differ significantly in terms of their means, ranges, and skewness. For example, lnDE has a mean of -5.6424, a standard deviation of 0.3856, and a skewness of 1.2690, indicating a moderately right-skewed distribution with slight kurtosis; lnGI has a skewness of -2.7312 and kurtosis of 30.8390, reflecting a highly peaked and left-skewed distribution—suggesting that green innovation in a few cities is exceptionally high, while most cities have relatively low levels. lnPM2.5 has a skewness of -2.8132, indicating that while most cities exhibit relatively good air quality, a small number face severe pollution levels.

The empirical data are collected at the prefecture-level city scale, encompassing a broad range of urban areas across China. Each observation corresponds to a distinct city, inherently characterized by spatial attributes. To explicitly model the spatial structure among these cities, a spatial weight matrix *W* is constructed based on first-order Rook contiguity. In this approach, two cities are defined as neighbors if they share a common administrative boundary. The contiguity relationships are derived from official geographic boundary shapefiles using GIS software, and the resulting binary adjacency matrix is row-standardized to facilitate interpretation. This spatial weight matrix is applied consistently throughout the spatial dependence diagnostics and econometric modeling.

In spatial econometrics, Moran’s I is a classical test used to detect whether a variable exhibits significant spatial autocorrelation. Spatial autocorrelation refers to the extent to which observations at nearby locations are statistically correlated [42]. The global Moran’s I statistic is computed to assess the spatial autocorrelation of each response variable: lnPM2.5, lnIWW, lnIDP, and $\ln ISO_{2}$.

Moran’s I is defined as [43]:

$$I=\frac{n}{W}\frac{\sum_{i=1}^{n}\sum_{j=1}^{n}w_{ij}(y_{i}-\bar{y})(y_{j}-\bar{y})}{\sum_{i=1}^{n}(y_{i}-\bar{y}{)}^{2}},$$ (24)

where *n* is the number of observations; *y*_*i*_ and *y*_*j*_ are the observed values for spatial units *i* and *j*; and $\bar{y}$ is the mean of variable *y*; *w*_*ij*_ denotes the (*i*,*j*)th element of the spatial weight matrix *W*, representing the spatial relationship between units *i* and *j*; and $W=\sum_{i=1}^{n}\sum_{j=1}^{n}w_{ij}$ is the total sum of all spatial weights.

The value of Moran’s I generally falls within the range of –1 to 1. A value of *I*>0 indicates positive spatial autocorrelation, meaning that neighboring regions tend to have similar values;*I*<0 implies negative spatial autocorrelation, where neighboring regions tend to have dissimilar or contrasting values; and I≈0 suggests the absence of significant spatial autocorrelation.

To evaluate the significance of spatial autocorrelation, a Monte Carlo simulation with 999 permutations was conducted to test Moran’s I statistic. Table 7 presents Moran’s I indices along with their corresponding significance test results for the response variables.

**Table 7:** Moran’s I indices and significance test results.

Variable	Moran’s I	Expected I	Variance	z-score	p-value	Spatial Correlation
lnPM2.5	0.8199	-0.0034	0.0022	17.4703	$2.41\times 10^{-68}$	Significantly Positive
lnIWW	0.4065	-0.0034	0.0022	8.6974	$3.40\times 10^{-18}$	Significantly Positive
lnIDP	0.2190	-0.0034	0.0022	4.7205	$2.35\times 10^{-6}$	Significantly Positive
$\ln ISO_{2}$	0.2477	-0.0034	0.0022	5.3292	$9.86\times 10^{-8}$	Significantly Positive

As shown in Table 7, the Moran’s I statistics for all response variables are substantially greater than zero, with corresponding z-scores exceeding conventional critical values and p-values well below 0.05. These results indicate significant positive spatial autocorrelation across all variables. In particular, lnPM2.5 shows a Moran’s I value of 0.8199, indicating an extremely high degree of spatial clustering. This suggests that PM2.5 concentrations in Chinese cities are highly spatially clustered, implying that neighboring cities tend to exhibit similar air quality levels. This spatial autocorrelation may stem from cross-regional pollutant diffusion, similarities in industrial structure, and insufficient environmental policy coordination among adjacent cities. For other variables, lnIWW exhibits a Moran’s I of 0.4065, significant at a high level, suggesting strong spatial autocorrelation in industrial wastewater discharge—likely linked to hydrological connectivity within river basins. Although the Moran’s I values for lnIDP and $\ln ISO_{2}$ (0.2190 and 0.2477, respectively) are relatively lower, they still reveal statistically significant positive spatial autocorrelation. This suggests that industrial particulate emissions and sulfur dioxide emissions also exhibit moderate regional clustering.

In addition, Moran’s I scatterplots were constructed to visualize spatial relationships. These scatterplots divide the observations into four quadrants: high-high, low-low, high-low, and low-high. The first and third quadrants represent positive spatial autocorrelation, while the second and fourth indicate negative spatial autocorrelation.

**Fig 7:**
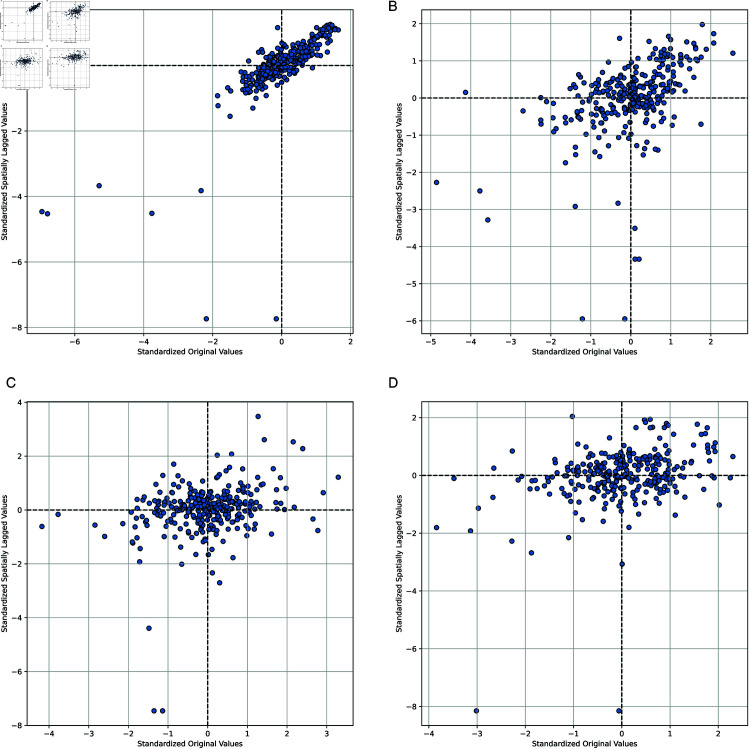
Moran’s I scatterplots of the response variables. (**a**) lnPM25; (**b**) lnIWW; (**c**) lnIDP; (**d**) $\ln ISO_{2}$.

Fig 7 shows the Moran’s I scatterplots for lnPM25, lnIWW, lnIDP, and $\ln ISO_{2}$, offering a visual interpretation of the spatial autocorrelation patterns for each variable.

As shown in Fig 7, the scatterplots of all response variables exhibit significant positive spatial autocorrelation, with most observations concentrated in the first and third quadrants. In particular, the scatterplot of lnPM2.5 displays the highest degree of concentration, further confirming its strong spatial clustering—suggesting that PM2.5 concentrations tend to be similar across neighboring cities. Similarly, the large number of observations in the first and third quadrants for lnIWW indicates strong spatial autocorrelation in industrial wastewater discharge, possibly linked to regional water systems and cross-boundary watershed pollution. Although the scatter distributions for lnIDP and $\ln ISO_{2}$ are more dispersed, the predominance of points in the first and third quadrants indicate that industrial smoke and dust emissions and sulfur dioxide emissions still exhibit certain positive spatial correlations. This pattern may be attributed to the inter-city spillover effects of industrial activities, such as shared energy structures or industrial configurations. Overall, Moran’s I scatterplots visually confirm the spatial autocorrelation patterns of the response variables, consistent with the statistical results reported in Table 7.

To further assess whether the response variables exhibit significant spatial dependence, both LM-Lag and LM-Error tests are conducted. The LM-Lag test evaluates the suitability of a spatial lag model, while the LM-Error test detects spatial dependence in the error terms. These diagnostic tests provide critical evidence for selecting an appropriate spatial econometric specification. The null hypothesis for the LM-Lag test is the absence of a spatial lag effect (i.e., ρ=0), whereas the LM-Error test assumes no spatial autocorrelation in the residuals (i.e., λ=0). The test results are shown in Table 8.

**Table 8:** LM-lag and LM-error test results.

Variable	LM-Lag Test			LM-Error Test		
	Statistic	p-value	Significance	Statistic	p-value	Significance
lnPM2.5	8.5129	0.0035	Significant	0.6733	0.4119	Not Significant
lnIWW	11.8851	$5.66\times 10^{-4}$	Significant	1.1623	0.2810	Not Significant
lnIDP	5.0266	0.0546	Marginal	0.8792	0.3484	Not Significant
$\ln ISO_{2}$	6.5289	0.0106	Significant	0.3884	0.5331	Not Significant

The LM-Lag test results indicate that most response variables exhibit significant spatial lag effects, whereas the LM-Error test results are generally not statistically significant. This suggests that spatial dependence among the environmental quality indicators is primarily reflected through lag effects, while spatial autocorrelation in the residuals is relatively weak. Specifically, the LM-Lag test results for PM2.5 concentration, industrial wastewater discharge, and industrial ${\text{SO}}_{2}$ emissions indicate significant spatial lag dependence—implying that environmental quality among neighboring cities is highly interdependent—likely due to cross-regional pollutant diffusion, interlinked economic activities, and inadequate environmental policy coordination. Although the LM-Lag test for industrial smoke and dust emissions is only marginally significant, it suggests that spatial dependence still exists and should not be entirely overlooked.

The empirical analysis begins with the application of the SAR model to examine the impact of digital economy development on urban environmental quality. The SAR model captures spatial dependence via a fixed spatial lag coefficient, assuming a homogeneous influence of neighboring observations across all regions. While this model offers a baseline framework for evaluating spatial interactions, it may fail to account for spatial heterogeneity that often characterizes real-world environmental processes. Therefore, the SAR model is first estimated and its performance evaluated, prior to introducing a more flexible specification. The SAR model employed in this study is specified as follows:

$$y_{i}=\;\rho \sum_{j=1}^{n}w_{ij}y_{j}+\beta_{0}+\beta_{1}\ln{DE}_{i}+\beta_{2}\ln{PD}_{i}+\beta_{3}\ln{GDPPC}_{i}+\beta_{4}\ln{FDI}_{i}\;+\beta_{5}\ln{SCI}_{i}+\beta_{6}\ln{GI}_{i}+\epsilon_{i},$$ (25)

Table 9 presents the estimation results of the SAR model applied to four environmental indicators. The results indicate that the estimated coefficients for the digital economy and green innovation are significantly negative in the models for lnPM2.5 and $\ln ISO_{2}$, aligning with theoretical expectations that technological progress reduces pollution. However, in the models for lnIWW and lnIDP, most explanatory variables display weak or insignificant effects. Specifically, in the lnIWW, the coefficients for the digital economy, population density, and foreign direct investment are not statistically significant, suggesting that the model fails to capture meaningful relationships. In the lnIDP, although several variables are statistically significant, the estimated signs and magnitudes vary considerably compared to those in other pollutant models. Furthermore, although the spatial lag parameter ρ is statistically significant across all models, its estimated magnitude varies considerably, indicating differing degrees of spatial dependence among the pollutants. These findings suggest that the SAR model, due to its constant spatial effect assumption, lacks the flexibility needed to capture spatial heterogeneity in environmental processes.

**Table 9:** Estimation results for the SAR model.

Parameter	lnPM2.5	lnIWW	lnIDP	$\ln ISO_{2}$
ρ	0.7150***	0.3350***	0.0930	0.1910**
Intercept	0.0938***	0.0000	0.0372***	0.1054***
lnDE	–0.0588	0.0375	–0.1770***	–0.0952
lnPD	0.2195***	0.0535	–0.0980**	–0.1314*
lnGDPPC	0.0488	0.0648*	0.2453***	0.2241***
lnFDI	–0.0086	–0.0303	–0.0101	–0.0694
lnSCI	0.0258	0.0201	–0.1043**	–0.0820
lnGI	–0.1013*	0.2365***	0.0552	0.0900

Based on the framework of the SSIVCAR model and the empirical data, the model is specified as follows:

$$y_{i}=\;g(u_{i}^{⊤}\alpha )\sum_{j=1}^{n}w_{ij}y_{j}+\beta_{0}+\beta_{1}\ln{DE}_{i}+\beta_{2}\ln{PD}_{i}+\beta_{3}\ln{GDPPC}_{i}+\beta_{4}\ln{FDI}_{i}\;+\beta_{5}\ln{SCI}_{i}+\beta_{6}\ln{GI}_{i}+\epsilon_{i},$$ (26)

where *y*_*i*_ denotes the response variable for city *i*, specifically one of $\ln{PM2.5}_{i}$, $\ln{IWW}_{i}$, $\ln{IDP}_{i}$, or $\ln{ISO_{2}}_{i}$.

The single-index varying coefficient $g(u_{i}^{⊤}\alpha )$ captures the spatially varying intensity of the lag effect. In this study, the spatial location *u*_*i*_ is defined using each city’s longitude and latitude, and $u_{i}^{⊤}\alpha$ represents the combined influence of spatial position on the lag intensity. By integrating *y*_*i*_ with the weighted average of its neighbors, $\sum_{j=1}^{n}w_{ij}y_{j}$, the model can effectively capture the spatial correlation characteristics of urban environmental quality.

We then estimate the parameters separately for the four response variables and denote significance levels (with ^*^ indicating significance, ** indicating high significance, and *** indicating extreme significance). Table 10 presents the estimation results for lnPM2.5, lnIWW, lnIDP, and $\ln ISO_{2}$.

**Table 10:** Estimation results for the SSIVCAR model.

Parameter	lnPM2.5	lnIWW	lnIDP	$\ln ISO_{2}$
$\alpha_{1}$	0.7485***	0.3361***	0.1326	0.8581***
$\alpha_{2}$	0.6631***	0.9418***	–0.9912**	0.5134**
Intercept	–2.2225**	–0.8268	–5.8610***	–8.3031***
lnDE	–0.0798**	–0.1557***	–0.3401***	–0.5123***
lnPD	0.0185	0.1933**	–0.2728**	–0.1320
lnGDPPC	0.1975***	0.3823***	0.5584***	0.6052***
lnFDI	–0.0290	0.0610	–0.0519	–0.0309
lnSCI	–0.0089	0.4931***	–0.0843	–0.0727*
lnGI	–0.0866***	–0.0184	–0.1298***	–0.1621***

Table 10 shows that the single-index parameters $\alpha_{1}$ and $\alpha_{2}$ are highly significant (with *p* < 0.001) for lnPM2.5 and lnIWW, indicating pronounced spatial lag effects for these pollutants. In addition, $\alpha_{2}$ is also significant in the model for $\ln ISO_{2}$ (*p* < 0.05). These results reflect a significant spatial association in environmental pollution across regions. Among the explanatory variables, the digital economy indicator (lnDE) has a significantly negative effect on all response variables—most notably on $\ln ISO_{2}$ (coefficient = –0.5123, *p* < 0.001)—suggesting that digital development plays an effective role in reducing industrial emissions and improving environmental quality. The effect of population density (lnPD) varies across different pollutants: it is significantly positive for lnIWW (*p* < 0.01), indicating that population concentration may exacerbate industrial wastewater discharge, whereas its significantly negative effect on lnIDP (*p* < 0.01) may be related to industrial restructuring and enhanced environmental governance. Per capita GDP (lnGDPPC) is significantly positively correlated in all models, suggesting that economic development is associated with greater environmental pressure. Finally, green innovation (lnGI) exhibits a significantly negative effect across all models, underscoring the important role of technological advancement in pollution mitigation.

In addition to the parameter estimates, we provide a statistical summary of spatial autoregressive coefficients, represented by the estimated values of $g(u_{i}^{⊤}\alpha )$ for the response variables lnPM2.5, lnIWW, lnIDP, and $\ln ISO_{2}$. The corresponding results are summarized in Table 11.

**Table 11:** Statistical description of spatial autoregressive coefficient.

Variable	Mean	Min	1st Quartile	Median	3rd Quartile	Max
lnPM2.5	0.1259	–0.2834	–0.0200	0.1261	0.2660	0.7665
lnIWW	0.1334	–0.7700	0.0260	0.1494	0.2567	0.5199
lnIDP	0.1484	–0.6950	0.0066	0.0932	0.1655	0.9210
$\ln ISO_{2}$	0.0651	–0.2476	–0.0216	0.0593	0.1550	0.9400

Table 11 reveals that spatial autoregressive coefficients of the response variables exhibit heterogeneity. For example, lnPM2.5 has a mean of 0.1259 and a median of 0.1261, ranging from –0.2834 to 0.7665, indicating that while the overall spatial lag effect for PM2.5 is weak, certain regions exhibit significant positive correlation—likely reflecting the diffusion characteristics of air pollution among urban agglomerations. Similarly, lnIWW has a mean of 0.1334 and ranges from –0.7700 to 0.5199, suggesting that while most areas exhibit positive lag effects, negative spatial dependence in some regions may be linked to disparities in local environmental governance. For lnIDP, the mean spatial autoregressive coefficient is 0.1484, with a maximum of 0.9210, indicating that industrial smoke and dust emissions are subject to strong spatial spillover effects in certain areas. Finally, $\ln ISO_{2}$ has a mean of 0.0651 and a median of 0.0593, but reaches a maximum of 0.9400, suggesting that sulfur dioxide emissions exhibit very strong spatial association in specific urban regions.

**Fig 8:**
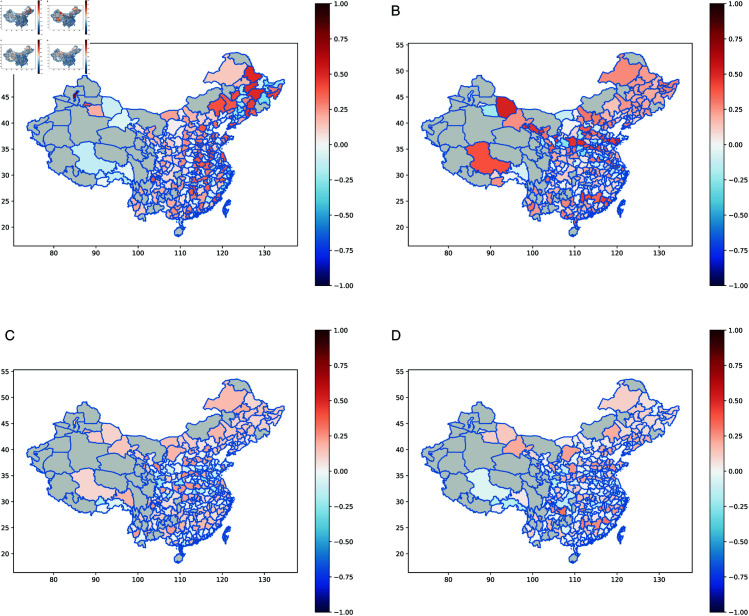
Spatial distribution of autoregressive coefficient for different response variables. (**a**) lnPM2.5; (**b**) lnIWW; (**c**) lnIDP; (**d**) $\ln ISO_{2}$. Map data source: GADM, available at https://geodata.ucdavis.edu/gadm/gadm4.1/shp/gadm41_CHN_shp.zip. Data is licensed under the CC BY License [45].

Furthermore, Fig 8 presents the spatial distribution of the estimated autoregressive coefficients for lnPM2.5, lnIWW, lnIDP, and $\ln ISO_{2}$.

Fig 8 illustrates the distribution of spatial autoregressive coefficient for lnPM2.5, lnIWW, lnIDP, and $\ln ISO_{2}$. The analysis shows that the spatial autoregressive coefficient of different environmental pollution indicators exhibits significant heterogeneity across regions, which reflects the combined influence of regional economic structures, industrial layouts, and pollutant dispersion mechanisms. For example, in Fig 8 (a), lnPM2.5 exhibits generally positive spatial correlation across most regions; northern regions (e.g., the Beijing-Tianjin-Hebei and Northeast regions) exhibit particularly high correlation (up to 0.7665), while southern regions show weaker or even negative correlations. This indicates that air pollution in northern regions exhibits marked cross-regional diffusion, likely driven by high heating demand, industrial emissions, and regional climatic conditions. In particular, the Beijing-Tianjin-Hebei region, characterized by high industrial concentration and pollutant emissions, faces severe cross-regional air pollution transmission. Therefore, establishing coordinated regional governance mechanisms in the Beijing-Tianjin-Hebei and surrounding areas is essential, along with promoting technological upgrades for key pollution sources, encouraging new energy substitution, and implementing coal-to-electricity conversion policies. Additionally, optimizing transportation systems and accelerating the phase-out of high-emission diesel vehicles will help reduce the cross-regional transmission of pollutants.

For lnIWW (Fig 8 (b)), the spatial autoregressive coefficient is more pronounced in coastal areas and certain economically developed inland cities, with a maximum value of 0.5199. By contrast, some central and western regions exhibit negative or insignificant spatial correlation. This suggests that industrial wastewater discharge in coastal areas has strong regional linkage, closely related to dense industrial clustering and well-developed water networks. For example, in the middle and lower reaches of the Yangtze River, industrial wastewater discharge affects not only local water quality but also has cascading impacts on upstream and downstream regions. These findings highlight the need for integrated basin-wide governance of water pollution, the promotion of advanced wastewater treatment technologies, and the implementation of recycled water utilization policies to mitigate cross-regional contamination.

The spatial autoregressive coefficient of lnIDP (Fig 8 (c)) is particularly high in energy-intensive cities in central and western regions (e.g., Shanxi, Shaanxi, Inner Mongolia), with a maximum value reaching 0.9210. The concentration of coal, steel, and other energy-intensive industries in these regions results in substantial emissions of industrial smoke and dust, which tend to follow fixed diffusion paths and generate cumulative regional impacts. In addition, some industrial cities in eastern coastal areas also exhibit high correlation intensity, indicating that industrial smoke and dust emissions adversely affect the air quality in adjacent cities. In response, central and western regions should prioritize industrial restructuring in high-energy-consuming sectors, accelerate supply-side reforms in the coal industry, and promote the adoption of low- and zero-emission technologies. Strengthening regional air quality monitoring networks and improving the precision of pollution source identification can further enhance the control of particulate matter pollution in key areas.

For $\ln ISO_{2}$ (Fig 8 (d)), the spatial autoregressive coefficient is highest in the Northeast, Bohai Rim, and East China regions, with a maximum value of 0.9400. These regions are characterized by concentrations of high-polluting industries such as chemicals and steel, leading to strong spatial linkage in sulfur dioxide emissions. In contrast, some industrial cities in central and western regions show lower sulfur dioxide emission intensities with weak spatial correlation due to limited atmospheric dispersion. To address these issues, regions with high pollution levels should accelerate the implementation of total ${\text{SO}}_{2}$ emission control policies. In particular, ultra-low emission retrofit technologies should be prioritized in the Northeast and Bohai Rim regions to reduce SO_2_ emissions from coal-fired power plants and the steel industry. Moreover, optimizing the regional distribution of heavy and chemical industries can gradually reduce the concentration of high-polluting sectors in a single area, thereby promoting cleaner production and improving regional air quality.

In summary, the spatial autoregressive coefficient distribution of different pollutants indicates that environmental governance must fully account for regional differences and pollutant diffusion patterns. In northern regions, PM2.5 control requires the establishment of cross-regional collaborative mechanisms and the promotion of clean energy substitution; in coastal economic zones, industrial wastewater management should focus on basin-wide coordination; and in central, western, and northeastern industrial areas, accelerating industrial upgrading and the promotion of clean technologies is essential to mitigate the impact of heavy-polluting industries. Targeted policies that avoid resource wastage while effectively reducing cross-regional pollutant diffusion provide a scientific basis for the comprehensive improvement of regional environmental quality.

To further investigate the impact of digital economy development on environmental quality, we examine a set of key variables that capture both environmental outcomes and socioeconomic characteristics. The response variables include lnPM2.5, lnIWW, lnIDP, and $\ln ISO_{2}$, representing different types of pollution indicators. The explanatory variables include the digital economy indicator, along with control variables including population density, per capita GDP, foreign direct investment, scientific expenditure, and green innovation. Table 5 presented earlier summarizes the descriptions and sources of these variables.

During the data preprocessing stage, all variables are log-transformed to reduce scale disparities and achieve more normalized distributions. Table 6 presents the descriptive statistics of the log-transformed variables.

Furthermore, we calculate the global Moran’s I for the response variables to assess their spatial autocorrelation, with the results presented in Table 7 and visualized via Moran’s I scatterplots (Fig 7). LM-Lag and LM-Error tests (Table 8) further confirm the presence of significant spatial lag effects, thereby supporting the application of a SAR model.

Based on the SSIVCAR model framework, we estimate the model specified in equation (26) for each response variable. Table 10 shows that the digital economy indicator (lnDE) has a significantly negative effect on environmental pollutants, whereas population density, per capita GDP, and green innovation exhibit heterogeneous effects across different pollutant indicators.

In addition, we analyze the spatial autoregressive coefficients, as summarized in Table 11 and illustrated in Fig 8. The results reveal significant heterogeneity in spatial correlation across different pollutants. For example, northern cities such as those in the Beijing-Tianjin-Hebei and Northeast regions exhibit high spatial correlation in PM2.5 concentrations, while coastal areas show strong positive spatial correlation in industrial wastewater discharge. Energy-intensive cities in central and western regions display very high spatial correlation in industrial smoke and dust emissions, and regions in the Northeast, Bohai Rim, and East China demonstrate pronounced spatial clustering in sulfur dioxide emissions.

These empirical findings indicate that digital economy development plays a significant role in improving environmental quality, although the magnitude and direction of its effects vary across pollutants and regions. The results underscore the need for targeted, region-specific environmental policies——for instance, establishing cross-regional coordination mechanisms in northern China to control PM2.5, promoting basin-wide coordination for water pollution control in coastal areas, and advancing industrial restructuring and the adoption of clean technologies in energy-intensive regions.

## Conclusion

This paper proposes a novel SSIVCAR model to address the limitations inherent in the SAR model. The SAR model assumes a uniform spatial correlation across all spatial units, which fails to capture the spatial heterogeneity commonly observed in applications. To overcome this shortcoming, we incorporate a non-linear single-index varying coefficient function, $g(u_{i}^{⊤}\alpha )$, into the classical framework. This formulation allows the spatial autoregressive coefficients to vary dynamically with the characteristics of individual spatial units, thereby providing a more accurate representation of complex spatial dependence structures.

Methodologically, we propose a joint estimation procedure that combines spline approximation with 2SLS to effectively estimate the model parameters. Through theoretical derivations, we derive explicit expressions for the parameter estimates. In addition, a series of Monte Carlo simulation experiments is designed to comprehensively evaluate the finite-sample performance of the model. The experimental results demonstrate that, compared to the SAR model, the new offers substantial improvements in capturing spatial heterogeneity and more accurately characterizes variations across spatial units. In comparative experiments, we further investigate the model’s performance under various spatial adjacency structures and different levels of random noise, with results indicating that the proposed model remains robust and broadly applicable across a range of conditions.

Finally, to validate the practical applicability of the proposed model, we apply it to examine the impact of digital economy development on environmental pollution in China. By selecting lnPM2.5, lnIWW, lnIDP, and $\ln ISO_{2}$ as representative indicators of environmental pollution, and incorporate digital economy indicators and relevant control variables into a comprehensive empirical framework. The study finds that digital economy development in China has a significant and spatially heterogeneous effect on various environmental pollution indicators. Specifically, for lnPM2.5 and lnIWW, digital economy development significantly reduces air pollution and industrial wastewater discharge, with more pronounced effects in economically developed coastal regions; for lnIDP and $\ln ISO_{2}$, the impact on industrial smoke and dust and sulfur dioxide emissions is more complex—exhibiting negative correlations in some regions, while in others it is shaped by industrial composition and the degree of policy coordination. Digital economy development contributes positively to pollution control by promoting green technological innovation, optimizing industrial structures, and improving resource use efficiency; however, its overall effectiveness remains constrained by regional economic development levels, industrial concentration, and the effectiveness of environmental governance policies.

Moreover, the empirical results of the SSIVCAR model further reveal the cross-regional characteristics and spillover effects of environmental pollution. The findings indicate that the diffusion of PM2.5 pollution is particularly pronounced in the Beijing-Tianjin-Hebei region, while industrial wastewater discharge exhibits strong regional interdependence in the Yangtze River Delta. These results suggest that environmental governance policies should be tailored to the spatial diffusion characteristics of pollutants by enhancing inter-regional collaboration and optimizing pollution control mechanisms. In addition, the findings highlight the importance of accounting for spatial heterogeneity in digital economy development to achieve the dual objectives of environmental improvement and sustained economic growth.

Future research may extend the application of this model to other domains or incorporate panel or time-series data to investigate time-varying spatial correlation effects, thereby providing deeper theoretical insights for spatial economics and evidence-based support for environmental governance policies.
